# Theoretical Study of Field-Free Switching in PMA-MTJ Using Combined Injection of STT and SOT Currents

**DOI:** 10.3390/mi12111345

**Published:** 2021-10-31

**Authors:** Shaik Wasef, Hossein Fariborzi

**Affiliations:** Electrical and Computer Engineering, King Abdullah University of Science and Technology, Thuwal 23955, Saudi Arabia; shaik.wasef@kaust.edu.sa

**Keywords:** combined spin-transfer torque (STT) and spin-orbit torque (SOT) switching, field like torque, damping like torque, magnetic tunnel junction

## Abstract

Field-free switching in perpendicular magnetic tunnel junctions (P-MTJs) can be achieved by combined injection of spin-transfer torque (STT) and spin-orbit torque (SOT) currents. In this paper, we derived the relationship between the STT and SOT critical current densities under combined injection. We included the damping–like torque (DLT) and field-like torque (FLT) components of both the STT and SOT. The results were derived when the ratio of the FLT to the DLT component of the SOT was positive. We observed that the relationship between the critical SOT and STT current densities depended on the damping constant and the magnitude of the FLT component of the STT and the SOT current. We also noted that, unlike the FLT component of SOT, the magnitude and sign of the FLT component of STT did not have a significant effect on the STT and SOT current densities required for switching. The derived results agreed well with micromagnetic simulations. The results of this work can serve as a guideline to model and develop spintronic devices using a combined injection of STT and SOT currents.

## 1. Introduction

**Figure 1:**
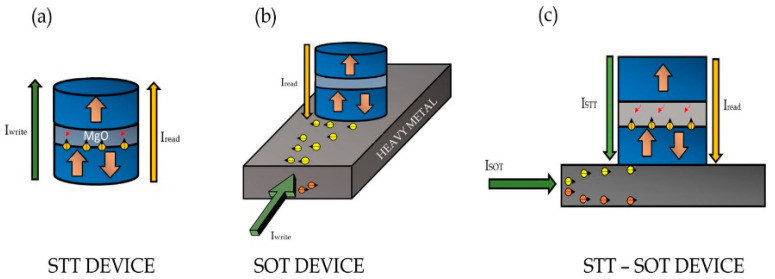
A schematic of the (**a**) spin-transfer torque (STT) device (**b**) spin-orbit torque (SOT) device and (**c**) STT-SOT device.

Information can be stored in ferromagnetic structures by the interaction between spin-polarized currents and magnetic moments. An magnetic tunnel junctions (MTJ) consists of a tunneling oxide layer (usually MgO) deposited between two ferromagnetic layers. Binary information is stored based on the relative orientation of the free layer (FL) to the reference layer (RL). An antiparallel (AP) orientation offers a high resistance and a parallel (P) orientation offers low resistance. Usually, the AP state is used to store bit “1” and the P state is used to store bit “0”. The AP or P state can be obtained by the interaction of the FL with spin-polarized charges. Depending on the mechanism of interaction, the magnetic storage devices can be classified into spin-transfer torque (STT) devices and spin-orbit torque (SOT) devices. In STT devices (Figure 1a), spin-polarized charges are generated via spin filtering from the RL of the MTJ. These charges can transfer their spin angular momentum to the FL, thereby exerting torque on its magnetization, which changes its magnetic orientation [1,2,3]. In SOT (Figure 1b), the magnetization switching in the free layer takes place due to the surface (Rashba effect) and bulk interactions (spin hall effect) caused by the attached heavy metal layer [4,5,6]. The magnetic reversal in the aforementioned mechanisms is due to the combined effects of DLT and FLT vector components [7,8,9,10]. In fact, the FLT component can affect the critical current required for switching in both STT and SOT devices [11,12]. Although commonly used, STT devices suffer from reliability and endurance issues caused by damage to the thin MgO tunneling layer. This happens because of the repeated tunneling of electrons, as the read and write paths are overlapped (both out of plane) [13,14]. In addition to this, an STT device suffers from incubation delay and, unlike SOT, does not realize sub-nanosecond switching [15]. On the other hand, an SOT device requires an external in-plane bias field for deterministic switching [16]. In order to overcome these constraints, devices operating under the combined effects of STT and SOT have been experimentally demonstrated [17]. The use of combined injection of STT and SOT currents provides a two-way advantage. The use of an STT current component facilitates complete magnetic reversal, which would otherwise require an external bias field in an SOT device. On the other hand, the SOT current component can provide lower switching time than a pure STT device. Due to these advantages, it was deemed necessary to comprehensively analyze the behavior of STT-SOT devices (Figure 1c). Although these devices have been extensively studied through macrospin simulations [18,19,20], their analysis under the influence of DLT and FLT has yet to be explored.

In this paper, we investigated the effects of combined injection of SOT $\left(J_{SOT}\right)$ and STT $\left(J_{STT}\right)$ current in P-MTJs with their individual DLT and FLT components under zero bias field. We first derived the critical STT density ($J_{critical}^{STT}$), required for switching in the absence of any SOT current. We then derived the relationship between the STT and SOT critical current densities when the ratio of the FLT to the DLT component of the SOT ($\beta_{SOT}$) was positive. We observed that, under combined injection, the critical SOT current density depended on damping constant and the magnitude of the FLT component of the STT current and the SOT current. We also noted that the critical STT and SOT current densities required for switching did not change considerably with the magnitude and sign of the FLT component of STT. However, they decreased with the increasing magnitude of FLT component of SOT. The derived results were verified with a micromagnetic model developed in OOMMF [21].

## 2. Landau–Lifshitz–Gilbert Equation with Spin-Transfer Torque (STT) and Spin-Orbit Torque (SOT) Terms

The magnetization dynamics of a ferromagnet under the influence of magnetic fields (internal and external) and spin currents can be described by the LLG equation with additional STT and SOT terms as given below [3].

(1) $$\frac{d\vec{m}}{dt}=-\gamma \left(\vec{m}\times \vec{H}\right)+\alpha \left(\vec{m}\times \frac{d\vec{m}}{dt}\right)+{\vec{\tau }}_{DL-SOT}+{\vec{\tau }}_{FL-SOT}+{\vec{\tau }}_{DL-STT}+{\vec{\tau }}_{FL-STT}$$

$${\vec{\tau }}_{DL-SOT}=-\gamma H_{SOT}\left(\vec{m}\times \left({\hat{p}}_{SOT}\times \vec{m}\right)\right)$$

$${\vec{\tau }}_{FL-SOT}=-\gamma \beta_{SOT}H_{SOT}\left(\vec{m}\times {\hat{p}}_{SOT}\right)$$

$${\vec{\tau }}_{DL-STT}=-\gamma H_{STT}\left(\vec{m}\times \left({\hat{p}}_{STT}\times \vec{m}\right)\right)$$

$${\vec{\tau }}_{FL-STT}=-\gamma \beta_{STT}H_{STT}\left(\vec{m}\times {\hat{p}}_{STT}\right)$$

Here, γ is the gyromagnetic ratio, $\beta_{STT}$ ($\beta_{SOT}$) is the ratio of the FLT to DLT of the STT (SOT), α is the damping constant, $\vec{m}$ is the unit vector which represents the magnetic orientation of the FL, ${\hat{p}}_{STT}$ and ${\hat{p}}_{SOT}$ are the spin polarization directions, and $H_{STT}$ and $H_{SOT}$ are the spin torque strengths of the STT and SOT, respectively, described as follows:

$$H_{STT}=\frac{\hbar \eta J_{STT}}{2eM_{s}t_{FM}}$$

$$H_{SOT}=\frac{\hbar \theta_{SHE}J_{SOT}}{2eM_{s}t_{FM}}$$

Here, e is the electron charge, ℏ is the reduced Planck’s constant, η is the spin polarization constant, $M_{s}$ is the saturation magnetization of the FL, $\theta_{SHE}$ is the spin hall angle, $t_{FM}$ is the thickness of the free layer, and $J_{STT}$ and $J_{SOT}$ are the STT and SOT charge current densities, respectively.

For simplicity, we ignored the effect of the stray fields of the RL on the FL. We also ignored the effects of the Oersted fields generated by the STT and SOT currents, as they only provided an initial misalignment in the FL magnetization and did not contribute significantly toward switching [22]. The analysis and the micromagnetic simulations (refer to methods: micromagnetic model) were developed based on Equation (1).

Unless otherwise specified, parametric values adopted in this work are mentioned in Table 1.

## 3. Results

### COMBINED STT-SOT Induced Switching in PMA-MTJ

**Figure 2:**
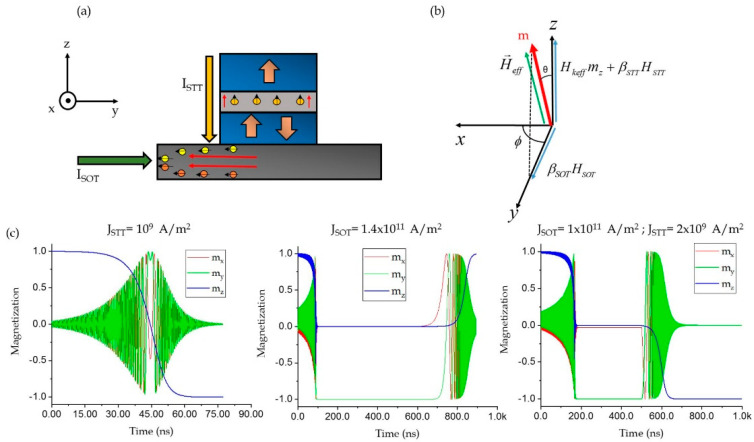
(**a**) Schematic of the STT-SOT device configuration used (**b**) Magnetization m relaxing to a point of equilibrium along the ${\vec{H}}_{eff}$ direction before reversal. (**c**) Magnetization dynamics of the FL in an STT, SOT and STT-SOT device.

In this section, we theoretically derived the relationship between the STT and SOT current densities under combined injection. The relationship was derived for FL switching from P to AP state. However, the same approach could be extended to obtain the relationship for switching from AP to P. The duration of the STT pulse in simulations was kept larger than the SOT pulse to promote deterministic switching [17]. As evident from Equation (1), the magnetic destabilization in these devices took place under the influence of an effective field (refer Figure 2b) given by

(2) $${\vec{H}}_{eff}=\vec{H}+\beta_{SOT}H_{SOT}{\hat{p}}_{SOT}+\beta_{STT}H_{STT}{\hat{p}}_{STT}$$

Switching when $\beta_{SOT}>0$ took place through precessions, since both STT and SOT directly compete with damping^12^ (refer Figure 2c). Thus, we were able to derive the relation between $J_{SOT}$ and $J_{STT}$ by linearizing the LLG equation. The magnetization dynamics of the FL under combined injection, as described by Equation (1), can be modified to the following form:

(3) $$\begin{array}{c} -\left(\frac{1+\alpha^{2}}{\gamma }\right)\frac{d\vec{m}}{dt}=\left(\vec{m}\times \vec{H}\right)+\alpha \left(\vec{m}\times \left(\vec{m}\times \vec{H}\right)\right)-H_{STT}\left(\alpha \beta_{STT}-1\right)\left(\vec{m}\times \left({\hat{p}}_{STT}\times \vec{m}\right)\right)+H_{STT}\left(\alpha +\beta_{STT}\right)\left(\vec{m}\times {\hat{p}}_{STT}\right)\\ -H_{SOT}\left(\alpha \beta_{SOT}-1\right)\left(\vec{m}\times \left({\hat{p}}_{SOT}\times \vec{m}\right)\right)+H_{SOT}\left(\alpha +\beta_{SOT}\right)\left(\vec{m}\times {\hat{p}}_{SOT}\right) \end{array}$$

Equation (3) can be linearized by converting the coordinate’s axes xyz to a new XYZ system where Z aligns with the direction of ${\vec{H}}_{eff}$ by using the rotation matrix R given by

$$R=\left(\begin{array}{ccc} \cos\theta \cos\phi & \cos\theta \sin\phi & -\sin\theta \\ -\sin\phi & \cos\phi & 0\\ \sin\theta \cos\phi & \sin\theta \sin\phi & \cos\theta \end{array}\right)$$

Here, θ and ϕ are the polar and azimuthal angles of the effective field when SOT and STT current approach their critical values (shown in Figure 2b). We linearized the LLG equation based on the assumption that the Z–component of magnetization remains unchanged at the beginning of the reversal and reversal occurs after small perturbations around the equilibrium direction. Thus, for simplification, we considered

$$\left\{ \begin{array}{l} M_{Z}=1\\ M_{Y}.M_{X}<<1\\ M_{X}^{2},M_{Y}^{2}=0 \end{array}\right.$$

Using the above assumptions Equation (3) can be modified into the following form

(4) $$\frac{1+\alpha^{2}}{\gamma }\left(\begin{array}{c} dM_{X}/dt\\ dM_{Y}/dt \end{array}\right)=M\left(\begin{array}{c} M_{X}\\ M_{Y} \end{array}\right)+G$$

Equation (4) has solutions of the form $M_{X},M_{Y}=A\exp\left(-\gamma \{[\pm i\sqrt{\left|M\right|-{\left(\mathrm{Trace}[M]/2\right)}^{2}}-\mathrm{Trace}[M]/2]t\}\right)$, where the real part in the exponential represents the time evolution of the oscillation amplitude. Thus, the realization of switching was based on the boundary condition of Trace [M] = 0. Hence, we obtained

(5) $$M_{11}+M_{22}=-2H_{keff}\alpha \cos^{2}\theta +H_{keff}\alpha \sin^{2}\theta +2H_{SOT}\left(1-\alpha \beta_{SOT}\right)\sin\phi \sin\theta +2H_{STT}(1-\alpha \beta_{STT})cos\theta =0$$

Substituting the values of θ and ϕ (from supplementary note 1), we first derived the critical switching current density ($J_{critical}^{STT}$) for STT-based switching, as follows:

(6) $$J_{critical}^{STT}=\frac{2et_{FM}M_{s}\alpha H_{keff}}{\hbar \eta (1-\alpha \beta_{STT})}$$

**Figure 3:**
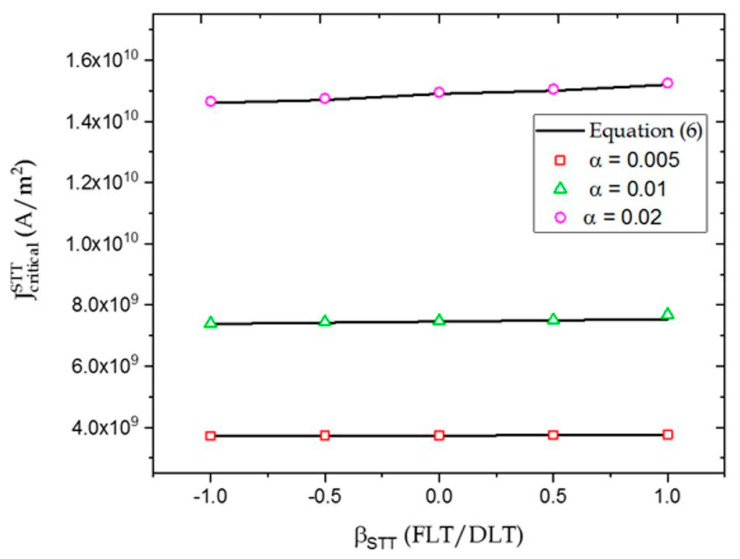
Dependence of $J_{critical}^{STT}$ on $\beta_{STT}$ for α = 0.005, 0.01, 0.02. The solid lines and symbols represent the results obtained from Equation (6) and micromagnetic simulations respectively.

From Equation (6), we observed that $J_{critical}^{STT}$ depended on the magnitude and sign of $\beta_{STT}$. $J_{critical}^{STT}$ did not change significantly with increase in $\beta_{STT}$, as shown in Figure 3. This result was consistent with observations made by Carpentieri et al. [24]. In addition to this, the rate of increase was relatively $J_{critical}^{STT}$, with $\beta_{STT}$ greater for larger values of α. The value of $\beta_{STT}$ depended on the properties of the materials [7,25,26,27,28,29,30] and was experimentally estimated to be between 0.01–0.1 for a CoFeB/MgO/CoFeB [29,30]. In this article, we used $\beta_{STT}$ values greater than the experimentally measured results to clearly show its effect. Here, a positive value of $J_{critical}^{STT}$ refers to the electrons moving from the FL to the RL.

Including the effects of SOT in Equation (5), we determined the relationship between the critical STT and SOT current densities, above which the P-MTJ switched from P-AP state as follows

(7) $$J_{SOT}=\frac{\sqrt{2}\sqrt{\alpha +\xi_{STT}J_{STT}(\alpha \beta_{STT}-1)}(1+\xi_{STT}J_{STT}\beta_{STT})}{\xi_{SOT}\sqrt{\beta_{SOT}(2+\alpha \beta_{SOT}-\xi_{STT}J_{STT}(\beta_{SOT}-2\beta_{STT}+\alpha \beta_{SOT}\beta_{STT}))}}$$

where $\xi_{STT}=\frac{\hbar \eta }{2et_{FM}M_{s}H_{keff}}$ and $\xi_{SOT}=\frac{\hbar \theta_{SHE}}{2et_{FM}M_{s}H_{keff}}$

**Figure 4:**
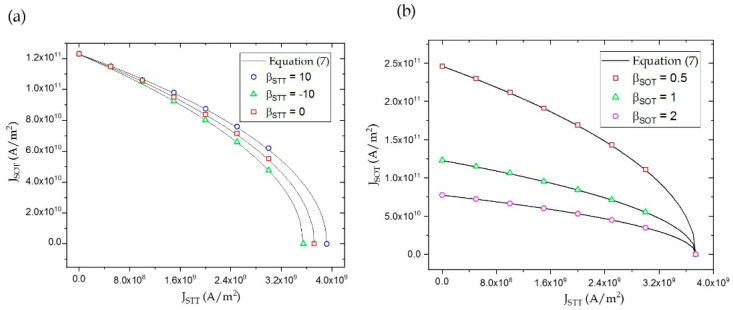
The solid line represents boundary Equation (7) above switching takes place from P to AP state (**a**), with changing $\beta_{STT}$ and (**b**) with increasing $\beta_{SOT}$. Symbols represent results obtained from micromagnetic simulations.

Equation (7) is valid only when $\beta_{SOT}>0$, since for $\beta_{SOT}=0$, switching did not take place entirely through precessions, although the STT always competed with the damping torque (refer to supplementary note 2, (Figure S1)). In the absence of $J_{STT}$, Equation (7) was consistent with results obtained by Tanuguchi.et al. [12]. As seen in Figure 4a, the critical current densities did not decrease appreciably, even for very large values of $\beta_{STT}$. However, their magnitudes decreased considerably with increasing values of $\beta_{SOT}$ (Figure 4b). This is because the FLT components of STT and SOT added to the effective field in the ${\hat{e}}_{z}$ direction and ${\hat{e}}_{y}$ direction, respectively (Equation (2)). Since the magnitude of $J_{STT}$ required for switching was lower than $J_{SOT}$, the contribution of its FLT component to the effective field was insignificant. Additionally, the FLT component of STT did not contribute toward a significant tilt in the magnetization. On the contrary, the FLT component of SOT was stronger, owing to the large SOT current density. As the FLT component of SOT was in-plane, it provided a larger tilt to the magnetization from its initial position, thereby reducing the individual critical current for switching. Hence, $J_{STT}$ and $J_{SOT}$, under combined injection, decreased appreciably for increasing values of $\beta_{SOT}$. Here, positive values of $J_{STT}$ refer to electrons flowing from FL to RL and positive values of $J_{SOT}$ refer to electrons flowing in the negative ${\hat{e}}_{y}$ direction (refer Figure 2a). It must be noted that deterministic switching took place only in the presence of combined STT and SOT and did not take place in the presence of SOT alone.

**Figure 5:**
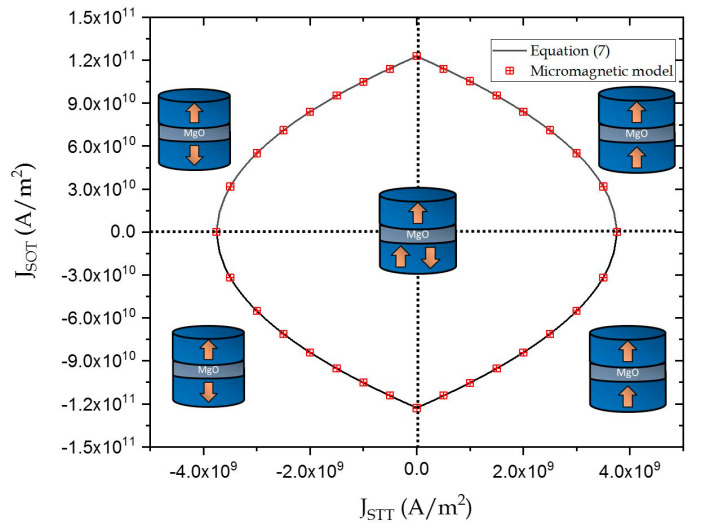
The solid line represents the boundary Equation (7). The symbols represent the results obtained from micromagnetic simulations.

SOT switching is symmetric in nature, since the final configuration of the FL is in-plane irrespective of the direction of current injection. Unlike SOT, STT-based switching is asymmetric, i.e., the magnitude of $J_{STT}$ for AP to P switching is lower than $J_{STT}$ required for P to AP switching. However, this inclusion was beyond the scope of this work. Figure 5 shows the boundaries separating the different regions of switching for parameters mentioned in Table 1. As seen in Figure 5, Equation (7) was consistent the experimental results obtained by Wang et al. [17].

## 4. Conclusions

In this work, we investigated the magnetic switching in MTJ devices under combined injection of Spin transfer torque (STT) and Spin orbit torque (SOT) currents. We included the effects of both the damping-like and field-like torque of the STT and SOT currents. We derived the relationship between the STT and SOT current densities when the ratio of the FLT to DLT component of the SOT was positive. We observed that the relationship between the critical SOT and STT current densities under combined injection depended on the damping constant and the magnitude of the FLT component of the STT current and the SOT current. However, unlike the FLT component of SOT, the magnitude and sign of the FLT component of STT had an insignificant effect on the STT and SOT current densities. The derived results were verified with a micromagnetic model.

## 5. Methods

### Micromagnetic Model

In this work, the micro-magnetic model was developed in OOMMF [21] based on Equation (1). Combined injection of STT and SOT was implemented using the “Oxs_SpinXferEvolve” extension module. The field-like torque components of STT and SOT were added as external magnetic fields with magnitudes depending on the individual injection currents. The duration of the STT current pulse was kept larger than the SOT to promote deterministic switching [17].

## Figures and Tables

**Table 1:** Input parameters used in this work unless otherwise specified.

Parameters	Numerical Values
γ	$17.32\times 10^{11}radT^{-1}s^{-1}$
α	0.005
η	0.33
$M_{s}$	$1.5\times 10^{6}A/m$ [23]
$t_{FM}$	1 nm [23]
$H_{Keff}$	540Oe [23]
$\theta_{SHE}(\beta -Ta)$	0.1 ^4^
${\hat{p}}_{STT}$	${\hat{e}}_{z}$
${\hat{p}}_{SOT}$	${\hat{e}}_{y}$
$\beta_{SOT}$	2
$\beta_{STT}$	1
A_exchange_	20 pJ/m
*T*_rise_ (*J*_STT_, *J*_SOT_)	0.5 ns
*T*_fall_ (*J*_STT_, *J*_SOT_)	0.5 ns

## Supplementary Materials

The following are available online at www.mdpi.com/article/10.3390/mi12111345/s1, Figure S1: Magnetic switching under combined injection of STT and SOT when $\beta_{SOT}=0$.
