# Supplementary material for: Theoretical Study of Field-Free Switching in PMA-MTJ Using Combined Injection of STT and SOT Currents
Source: Micromachines (Basel). 2021 Oct 31;12(11):1345. doi: 10.3390/mi12111345 (PMC8619685; doi:10.3390/mi12111345)
Supplement: Supplementary file 1 [file micromachines-12-01345-s001.zip › micromachines-1378454-supplementary.pdf]

Supplementary Materials: Theoretical Study of Field-Free Switching in PMA-MTJ Using Combined Injection of STT and SOT Currents

Shaik Wasef and Hossein Fariborzi *

Electrical and Computer Engineering, King Abdullah University of Science and Technology, Thuwal 23955, Saudi Arabia; shaik.wasef@kaust.edu.sa
* Correspondence: hossein.fariborzi@kaust.edu.sa

Note 1. Estimating the Polar (θ) and Azimuthal (ϕ) Angles of Equilibrium Direction of Magnetization under Combined Injection

Using Equation (2) the equilibrium direction of magnetization when aligned along the effective field can be calculated using the following equations

$$\begin{cases} gm_x = 0 \\ gmy = \beta_{SOT} H_{SOT} \\ gmz = H_K m_Z + \beta_{STT} H_{STT} \end{cases} \qquad \text{(A.1)}$$

Here g is a proportionality constant. From the first 2 equations of equation (A.1) we calculate $\phi = 90^o$ and θ can be calculated by approximating it as a small angle. With this approximation, θ is given as follows

$$\theta = \frac{\beta_{SOT} H_{SOT}}{H_{Keff} + \beta_{STT} H_{STT}}$$

Note 2. Switching under Combined Injection of STT and SOT when $\beta_{SOT} = 0$

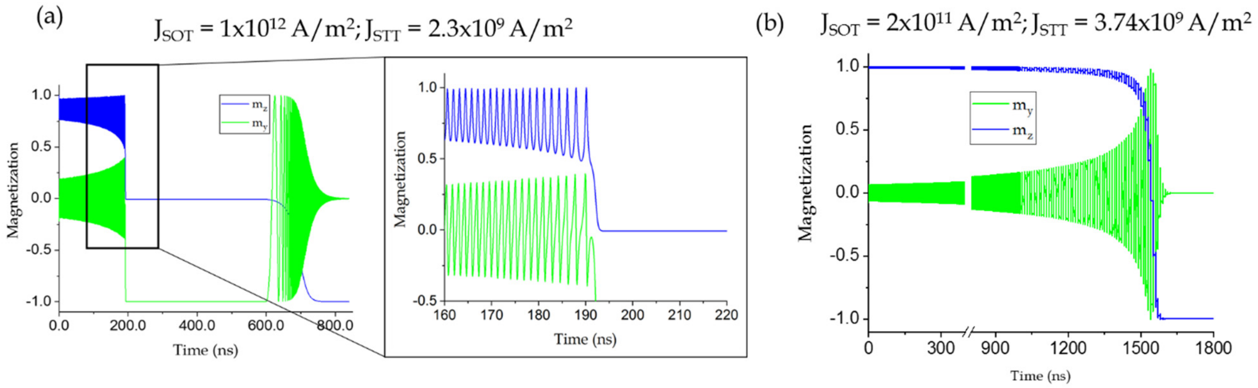

Figure S1. Magnetic switching under combined injection of STT and SOT when $\beta_{SOT} = 0$.

As seen in Figure S1a when $\beta_{SOT} = 0$, the switching mechanism is complex when the SOT dominates the STT . In such a scenario, the switching does not take place in one precession unlike the case when only SOT is present [1,2]. This is because in the absence of STT , when the SOT current is less than its critical value the magnetization saturates to a stable point above the x-y plane through the damping torque. On the other hand, the STT compensates for the damping torque and induces growing precessions. When $\beta_{SOT} = 0$, the SOT directly competes with the precessional torque of the effective field [2].The magnitude of the effective field decreases with the decreasing z – component of

magnetization (refer Equation (6) of the main text) . At a certain point during the growing precessions caused by the STT, the magnitude of SOT will be higher than precessional torque of the effective field and thus the magnetization switches to the in-plane state.

When the STT dominates the SOT the switching take place entirely through precessions since the SOT just provides an initial tilt to the magnetization (refer figure S1 (b))

References

[1] Lee, K.S.; Lee, S.-W.; Min, B.-C. and Lee, K.-J. Threshold current for switching of a perpendicular magnetic layer induced by spin Hall effect. *Appl. Phys. Lett.* **2013**, *102*, 112410.

[2] Taniguchi, T.; Mitani, S. and Hayashi, M. Critical current destabilizing perpendicular magnetization by the spin Hall effect, Phys. Rev. *B* **2015**, *92*, 024428.
